# *Aspergillus* sensitization associated with current asthma in children in the United States: an analysis of data from the 2005-2006 NHANES

**DOI:** 10.4178/epih.e2022099

**Published:** 2022-10-28

**Authors:** Hui-Ju Wen, Shu-Li Wang, Ming-Chieh Li, Yue Leon Guo

**Affiliations:** 1 National Institute of Environmental Health Sciences, National Health Research Institutes, Miaoli, Taiwan; 2 Institute of Earth Science, Academia Sinica, Taipei, Taiwan; 3 Department of Public Health, National Defense Medical Centre, Taipei, Taiwan; 4 Department of Safety, Health, and Environmental Engineering, National United University, Miaoli, Taiwan; 5 Department of Health Promotion and Health Education, College of Education, National Taiwan Normal University, Taipei, Taiwan; 6 Department of Environmental and Occupational Medicine, National Taiwan University (NTU) College of Medicine and NTU Hospital, Taipei, Taiwan; 7 Institute of Environmental and Occupational Health Sciences, National Taiwan University College of Public Health, Taipei, Taiwan

**Keywords:** IgE, Asthma, Children, *Aspergillus*

## Abstract

**OBJECTIVES:**

This study investigated the association between allergen sensitization and current asthma in children in the United States using data from the 2005-2006 National Health and Nutrition Examination Survey (NHANES).

**METHODS:**

Children who participated in the 2005–2006 NHANES, aged 6 years to 19 years, were included in this study. A structured questionnaire was used to assess asthma status (without asthma, asthma in remission, or current asthma). Nineteen specific immunoglobulin E (sIgE) levels were measured using the Pharmacia Diagnostics ImmunoCAP 1000 System (Kalamazoo, MI, USA). A machine-learning method was applied to select important sIgEs related to childhood asthma. Multivariate regression analysis was used to test this hypothesis.

**RESULTS:**

In total, 2,875 children were recruited. The prevalence of ever having asthma and current asthma was 16.5% and 5.6%, respectively. Six sIgE levels were found to contribute to asthma using bootstrap forest selection. After adjusting for the child’s sex, age, and family income, children with double the sIgE levels of *Dermatophagoides farinae*, dogs, and *Aspergillus* were more likely to have current asthma than children without asthma (odds ratio [95% confident interval]: 1.11 [1.04 to 1.19], 1.30 [1.16 to 1.46], and 1.55 [1.39 to 1.72], respectively).

**CONCLUSIONS:**

Our findings suggest that allergen sensitization, especially to *Aspergillus*, is associated with current asthma in children. Strategies to reduce sensitization may help prevent and manage asthma.

## INTRODUCTION

Asthma is a major non-communicable disease [1]. Globally, data from 2019 revealed that asthma affected an estimated 262 million individuals, resulted in 461,000 deaths, and was responsible for 21.6 million disability-adjusted life years [2,3]. Asthma is one of the most common chronic respiratory inflammatory diseases in childhood. It exerts profound effects on the quality of life and socioeconomic consequences in children and their families [4]. According to the National Health Interview Survey (NHIS) in 2000-2019, the prevalence of lifetime asthma in the United States was more than 10% (range, 10.5-14.0%) in children aged < 18 years [5]. In contrast, the prevalence of current asthma is approximately 7.0-9.6%. Of those with asthma, more than 40% (range, 44.3-57.9%) of children (< 18 years old) reported asthma attacks in the 2011-2019 NHIS annual survey [5].

Asthma is a multifactorial disorder. Environmental and hereditary factors contribute to asthma development. Sensitization to allergens is a major factor in allergic diseases and is also associated with the occurrence of asthma [6]. The quantity of specific immunoglobulin E (sIgE) in the serum is considered a biomarker of antigen exposure and sensitization. Higher sIgE levels for cat and dog dander were strongly associated with the prevalence, severity, and persistence of asthma in Swedish children, aged 19 years [7]. A higher prevalence of uncontrolled asthma symptoms was found in patients with *Aspergillus* sensitization than in those who were not sensitized [8]. Matsui et al. [9] reported a high correlation between sIgEs and dust allergen levels for dust mites, mice, and cockroaches. Children with higher sIgE levels for cats, dust mites, and mice were also observed to have poorer lung function, a higher fractional exhaled nitric oxide concentration, a higher blood eosinophil count, and an increased risk of exacerbations or hospitalization for asthma [9]. Utilizing sIgEs as a predictor of asthma morbidity could be useful for physicians in the management and treatment of asthma.

The persistence of asthmatic attacks remains an important public health issue. However, the role of sensitization to allergens on asthma persistence remains unknown. This investigation aimed to evaluate the association between allergen sensitization and current asthma in children by using data from the National Health and Nutrition Examination Survey (NHANES).

## MATERIALS AND METHODS

### Participants

The NHANES is a nationally representative survey conducted in the United States by the National Center for Health Statistics (NCHS) of the Centers for Disease Control and Prevention [10]. NHANES uses a complex, multistage, probability sampling design to recruit representative participants of the United States population. In 1999, the survey became a perpetual program with surveys conducted bi-annually.

Among the NHANES data, sIgEs were measured only in the 2005-2006 NHANES. Nine sIgEs were assessed in participants aged less than 6 years, and 19 sIgEs were assessed in participants aged ≥ 6 years. The present study, therefore, included participants aged 6-19 years in the 2005-2006 NHANES survey.

### Asthma classification

A medical history questionnaire was used to identify children who had asthma. The asthma status was classified according to the following three questions: question 1 (Q1). “Has a doctor or other health professional ever told you that you have asthma?”, Q2. “Do you still have asthma?”, and Q3. “During the past 12 months, have you had an episode of asthma or an asthma attack?”. A “no” response to Q1 was considered as never having asthma (never group). A “yes” response to Q1 was considered as ever having asthma (ever group). “Yes” responses to Q1, Q2, and Q3 were considered as having current asthma (current asthma group).

### Specific immunoglobulin E measurement

Serum samples were analyzed to identify the 19 sIgEs using the Pharmacia Diagnostics ImmunoCAP 1000 System (Kalamazoo, MI, USA). The IgE antibodies measured included *Aspergillus*, dog, *Alternaria*, *Dermatophagoides farina* (Der F), *Dermatophagoides pteronyssinus* (Der P), cat, ragweed, Bermuda grass, rye grass, thistle, oak, birch, mouse, peanut, cockroach, shrimp, rat, milk, and egg. The sIgE lower and upper limits of detection were 0.35 IU/mL and 1,000 IU/mL, respectively. sIgE concentrations below the lower limit of detection (LOD) were replaced with LOD/√2.

### Statistical analysis

All statistical analyses were performed using the JMP version 13.0 (SAS Institute Inc., Cary, NC, USA). We used the “Predictor Screening” platform of JMP based on bootstrap forest analysis (default 100 trees), a machine learning method, to select the important sIgEs for ever having asthma. sIgEs with an average contribution portion of > 0.05 were selected for further statistical analysis after conducting the bootstrap forest analysis 5 times (Supplementary Material 1). Correlations between selected sIgEs and asthma were assessed using Spearman correlation coefficients. Backward stepwise regression was applied to select significant sIgEs in the multivariate regression model. We entered all selected sIgEs in the stepwise regression model and dropped them 1 by 1, starting with the highest p-values until all the p-values were < 0.05. The sIgE levels were log2-transformed owing to their skewed concentration distribution, and the effect was assessed based on the doubling of sIgE levels. A p-value of < 0.05 in a 2-sided test was considered statistically significant.

### Ethics statement

The use of the NHANES data was approved by the NCHS Research Ethics Review Board (protocol #2005-06). All the participants provided written informed consent before participating.

## RESULTS

**Figure 1.**
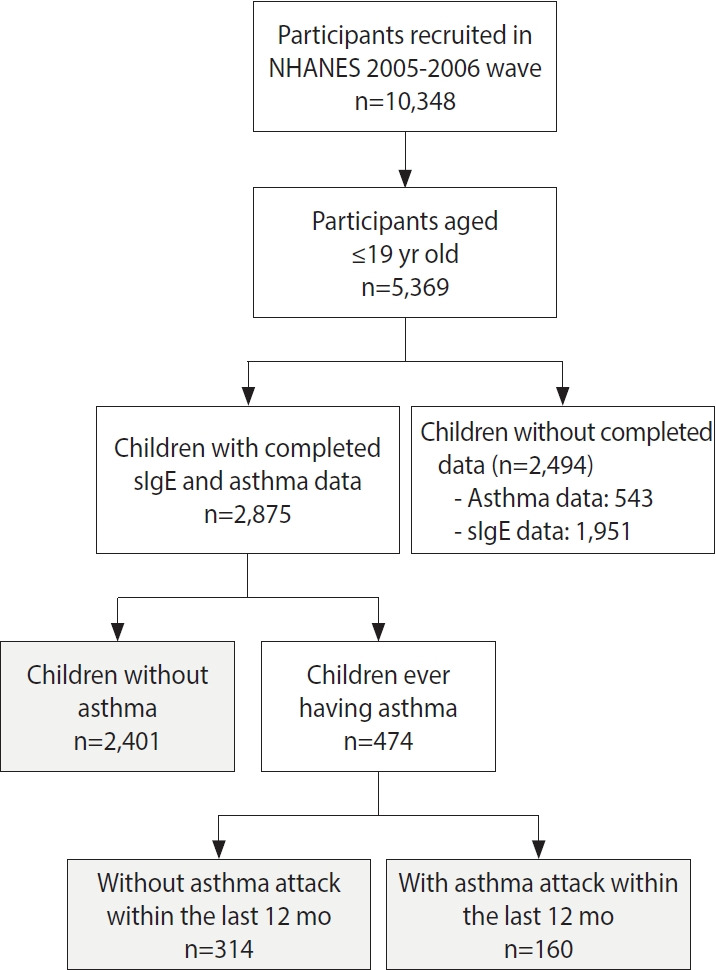
Flow chart of participants’ recruitment. NHANES, National Health and Nutrition Examination Survey; sIgE, specific immunoglobulin E.

A total of 2,875 children aged 6-19 years were included in this study (Figure 1). The prevalence of children who had been diagnosed with asthma and those with current asthma was 16.5% and 5.6%, respectively. The prevalence of asthma was higher in boys than in girls (p= 0.049). We then used sex-specific and age-specific 85th percentile of body mass index (BMI) values as the cut point for indicating overweight/obesity in children (Supplementary Material 2). Children with asthma had higher BMI values than those without asthma (p= 0.003). The prevalence of overweight or obesity was also higher in children with asthma than in those without asthma (p= 0.030). A family income ≥ US$55,000 was more common among children in the current asthma group than in the never group (p= 0.025) (Table 1). The distribution of the 19 sIgE levels in children is shown in Table 2. The percentage of sIgE levels above the LOD ranged from 1.7% to 26.5%.

The average contribution of the 19 sIgEs to ever having asthma in children, as determined by 5 bootstrap forest analyses, is shown in Supplementary Material 1. The sIgE with the largest contribution portion to ever having asthma in children was the *Aspergillus* IgE antibody (0.17), followed by antibodies from dogs (0.16) and *Alternaria* (0.15). Six sIgE levels with contribution portion of more than 0.05 were selected for further analysis: specifically, Der F, Der P, cat, dog, *Aspergillus*, and *Alternaria* (Supplementary Material 1). A high correlation was found among the 6 selected sIgEs, especially for Der F and Der P (correlation= 0.920), dog and cat (correlation = 0.657), and *Aspergillus* and *Alternaria* (correlation= 0.747), respectively (Supplementary Material 3). The percentage of results under the LOD (%< LOD) for the 6 selected sIgE levels among the 3 groups is shown in Supplementary Material 4. The percentage of results above the LOD in the 6 selected sIgEs was higher in the current asthma group than in the other 2 groups (p< 0.001). Higher levels of sIgEs were measured in children in the current asthma group and the ever group than in the never group, respectively (p< 0.001) (Supplementary Material 5).

Table 3 shows the associations between sIgE levels and asthma status in children. After adjusting for the child’s sex, age, overweight/obesity, and family income, children with double the levels of sIgEs had an increased risk of current asthma, with adjusted odds ratios (aORs) of 1.22 (95% confidence interval [CI], 1.15 to 1.29) for Der F, 1.20 (95% CI, 1.14 to 1.27) for Der P, 1.39 (95% CI, 1.29 to 1.50) for cats, 1.59 (95% CI, 1.45 to 1.74) for dogs, 1.39 (95% CI, 1.30 to 1.49) for *Alternaria*, and 1.75 (95% CI, 1.58 to 1.93) for *Aspergillus* (Table 3). Significant increases in the aORs of sIgEs were also found in children with asthma (ever group) compared to those in children without asthma (never group) (Table 3).

Backward stepwise regression analysis was then applied to select significant sIgE levels relative to asthma status. The results after adjusting for covariates are presented in model 2 of Table 3. Children with double the levels of Der P, dog, and *Aspergillus* IgE had an increased risk of asthma (aOR, 1.09; 95% CI, 1.05 to1.14 for Der P, aOR, 1.25; 95% CI, 1.15 to 1.35 for dogs, and aOR, 1.39; 95% CI, 1.28 to 1.50 for *Aspergillus*, respectively). Children with double the levels of Der F, dog, and *Aspergillus* IgE also had an increased risk of current asthma (aOR, 1.11; 95% CI, 1.04 to 1.19 for Der F; aOR, 1.30; 95% CI, 1.16 to 1.46 for dogs; and aOR, 1.55; 95% CI, 1.39 to 1.72 for *Aspergillus*, respectively). The crude ORs (OR1) for the 3 groups are shown in Supplementary Material 6.

Compared to children with asthma in remission, children with double the levels of sIgE had an increased risk of current asthma (aOR, 1.09; 95% CI, 1.02 to 1.17 for Der F; aOR. 1.15; 95% CI, 1.05 to 1.26 for cats; aOR, 1.19; 95% CI, 1.08 to 1.32 for dogs; aOR, 1.15; 95% CI, 1.06 to 1.25 for *Alternaria*; and aOR, 1.22; 95% CI, 1.10 to 1.35 for *Aspergillus*, respectively) (Table 4). The aOR of per interquartile range increase in sIgE levels is also shown in Table 4. After backward stepwise regression analysis, children with double the levels of cat and *Aspergillus* IgE had an elevated risk of current asthma (aOR, 1.12; 95% CI, 1.02 to 1.24 for cats and aOR, 1.19; 95% CI, 1.08 to 1.33 for *Aspergillus*) (Table 4). The crude ORs (OR2) between children with asthma in remission and with current asthma is shown in Supplementary Material 6.

## DISCUSSION

Allergen sensitization is known to be associated with the occurrence and onset of asthma. In the present study, we found that children with higher sIgE levels, especially those with sIgE against *Aspergillus*, showed an increased risk of current asthma. Children with asthma also had higher sIgE levels than those without asthma.

The imbalance of Th1 and Th2 immune responses has been suggested as a biological mechanism of allergic diseases. Allergen-specific responses in asthma are attributed to a skew towards a Th2 phenotype, with elevated levels of serum interleukin-4 and IgE. Elevated IgE levels are biomarkers of allergen sensitization. Patients who are sensitized to specific allergens can be identified through sIgE measurements. Therefore, testing sIgE levels may be beneficial for disease prevention and management to avoid allergen exposure. Asthmatic children sensitized to molds, dust mites, or animal dander were observed to have an increased risk of current asthma in the present study. Among the measured sIgE levels, the effect of *Aspergillus* sIgE on current asthma was the most significant, after adjusting for other sIgE levels. Byeon et al. [11] observed that sensitization to molds increased airway hyper-responsiveness and was associated with lower lung function in children with asthma. Rajagopal et al. [8] reported that asthma severity may be associated with fungal sensitization. Among asthmatic patients, a higher proportion of patients with uncontrolled symptoms were observed in those with *Aspergillus* sensitization. Frequent exacerbations and a greater corticosteroid requirement were also found in asthmatic children with *Aspergillus* sensitization [12].

Moreover, allergic bronchopulmonary aspergillosis (ABPA), a pulmonary disease caused by hypersensitivity to *Aspergillus fumigatus* (AF), is an important etiology for patients with poorly controlled asthma. In addition to asthma exacerbation, the pathological characteristics of ABPA include bronchial mucoid impaction, eosinophilic pneumonia, and bronchocentric granulomatosis [13]. Asthmatic children with ABPA have poorer lung function than those without ABPA [14]. More brownish sputum was also observed in children with ABPA [15]. Patients who still have poorly controlled asthma after receiving conventional treatment for asthma and are sensitized to *Aspergillus* should be suspected of having ABPA. The diagnostic criteria of ABPA are: (1) the presence of asthma, cystic fibrosis, or chronic obstructive pulmonary disease; (2) positive results for proximal bronchiectasis; (3) a positive AF skin prick test or AF sIgE >0.35 IU/mL; and (4) total serum IgE >417 IU/mL [16]. In our study, the prevalence of *Aspergillus* sIgE >0.35 IU/mL and total IgE >417 IU/mL was 9.4% (n=15) in the current asthma group. Importantly, these children may potentially have an allergic condition similar to ABPA and require further evaluation.

Molds are common environmental organisms found in our surroundings. According to a nationally presentative birth cohort in Taiwan, indoor molds are a predictor of asthma development in children [17]. *Aspergillus*, *Penicillium*, and basidiospores are among the fungal species associated with asthma and atopy in schoolchildren [18]. High ambient levels of *Cladosporium* spores were also associated with reduced lung function in schoolchildren in a longitudinal follow-up study [19].

The house dust mite (HDM) is another major allergen that induces allergic diseases, and Der F and Der P are the most important sources of HDM exposure. A relationship between Der F and Der P sensitization and current asthma was found in this present study. In a longitudinal study in Taiwan, early sensitization to HDM was associated with an increased risk of developing asthma and abnormal lung function in 7-year-old children [20]. In a birth cohort study in Germany, Posa et al. [21] observed that early exposure to Der P allergens might predict current or future asthma in children aged 6 years to 20 years. Sensitization to Der F was also associated with decreased post-decongestant nasal volume in Korean children [22].

We also found that dog and cat dander were associated with asthma in children. Cats and dogs are beloved household pets [23]. However, dander is a common allergen for patients who are prone to allergies. Decreased lung function has been observed in children sensitized to dog dander [22]. In a prospective, population-based cohort study in Sweden, Perzanowski et al. [7] observed that sIgE levels for cat and dog dander were strongly associated with cases of physicians-diagnosed asthma and were related to the persistence and severity of asthma in 19-year-old children.

Although we found that sensitization to molds, animal dander, and dust mites was associated with current asthma, the species of these allergens are diverse [24-26], and the results need further confirmation in order to precisely help sensitized patients avoid allergens in daily life or receive proper therapy.

A major strength of this study is that it included members of the general population based on a multistage, probability sampling design, which can enhance the population representativeness of 6-year-old to 19-year-old children in the United States. Moreover, using a machine learning method allowed us to efficiently determine the significant sIgEs for childhood asthma when considering all sIgE measurements, as compared to the classic epidemiological method of multiple testing individually.

Nonetheless, the present study has some limitations. First, it is difficult to establish a temporal relationship between exposure and outcome in a cross-sectional study design using the NHANES. However, allergen sensitizations (sIgE) were assessed through allergen-antibody binding using enzyme-linked immunosorbent assays. The biological pathogenic pathway involves sensitization to allergens and disease occurrence, but not the opposite route. Therefore, the relationship between exposure and disease can be established. Second, asthma status was based on a patient’s recall and a physician’s diagnosis of asthma. We did not inquire whether this diagnosis was made by a pediatrician, pulmonologist, or general practitioner. The misclassification of childhood asthma is a cause for concern. However, the diagnoses were most likely made by members of those specialties, which in turn, may have reduced the level of misclassification. Third, hereditary factors are major risk factors for asthma. The lack of information from the NHANES about the family history of allergic diseases meant that we could not adjust for this factor in the model. Moreover, other factors related to sIgE levels, such as the microbiota content [27], were not measured or adjusted for in the present study. This may have reduced the effect of sIgE levels on childhood asthma. Fourth, only 1 blood sample was collected for sIgE measurements, which may not represent the long-term status of children. However, atopic sensitization to allergens is innate and sIgE levels are generally stable. Hence a single measurement can still represent a child’s allergen sensitization.

In conclusion, the findings of this study suggest that allergen sensitization, especially to *Aspergillus*, was associated with current asthma in children. Avoiding allergen exposure is beneficial for preventing and managing asthma.

## Figures and Tables

**Table 1.** Characteristics of participants aged 6-19 years in the 2005-2006 NHANES survey

Characteristics		All	Asthma			p-value^1^	
			Never	Ever	Current^2^	Never vs. ever	Never vs. current
Total		2,875 (100)	2,401 (83.5)	474 (16.5)	160 (5.6)		
Age (yr)		13.30±3.88	13.29±3.91	13.62±3.67	12.75±3.69	0.141	0.065
Sex							
	Boys	1,428 (49.7)	1,173 (48.9)	255 (53.8)	88 (55.0)	0.049	0.132
	Girls	1,447 (50.3)	1,228 (51.1)	219 (46.2)	72 (45.0)		
BMI (kg/m^2^)		22.63±6.35	22.47±6.28	23.45±6.65	22.54±6.10	0.003	0.737
	Obesity/overweight	419 (14.6)	335 (14.0)	84 (17.7)	23 (14.4)	0.030	0.870
	No	2,444 (85.0)	2,058 (85.7)	386 (81.4)	136 (85.0)		
	Missing	12 (0.4)	8 (0.3)	4 (0.8)	1 (0.6)		
Family income (US$)							
	<25,000	903 (31.4)	759 (31.6)	144 (30.4)	40 (25.0)	0.198	0.025
	25,000-55,000	874 (30.4)	735 (30.6)	139 (29.3)	47 (29.4)		
	≥55,000	978 (34.0)	796 (33.2)	182 (38.4)	71 (44.4)		
	Missing	120 (4.2)	111 (4.6)	9 (1.9)	2 (1.3)		

Values are presented as number (%) or mean±standard deviation. NHANES, National Health and Nutrition Examination Survey; BMI, body mass index.

1 Calculated by the chi-square test for categorical variable and the Wilcoxon rank sum test for continuous variables.

2 Current: asthma attack within 12 months.

**Table 2.** Distribution of sIgE levels in children aged 6 to 19 years (n=2,875)

sIgE (IU/mL)	n	GM	AM±SD	Median	IQR	Range	%>LOD
Der F	2,874	0.49	6.08±39.86	0.25	0.25-0.25	0.25-1,000	24.0
Der P	2,875	0.51	6.98±43.18	0.25	0.25-0.25	0.25-1,000	24.6
Dog	2,875	0.33	1.23±12.87	0.25	0.25-0.25	0.25-558	16.8
Cockroach	2,875	0.35	1.30±7.82	0.25	0.25-0.25	0.25-204	15.4
Cat	2,875	0.34	1.72±14.52	0.25	0.25-0.25	0.25-476	13.7
Mouse	2,871	0.27	0.57±5.23	0.25	0.25-0.25	0.25-196	2.4
Rat	2,875	0.26	0.33±1.65	0.25	0.25-0.25	0.25-73.6	1.7
Peanut	2,875	0.32	1.05±9.10	0.25	0.25-0.25	0.25-413	11.9
Milk	2,855	0.27	0.31±0.58	0.25	0.25-0.25	0.25-25.2	8.8
Shrimp	2,875	0.29	0.82±7.71	0.25	0.25-0.25	0.25-322	8.8
Egg	2,875	0.26	0.28±0.40	0.25	0.25-0.25	0.25-15.8	3.4
Rye grass	2,875	0.59	9.19±48.75	0.25	0.25-0.45	0.25-1,000	26.5
Bermuda grass	2,875	0.47	4.68±30.59	0.25	0.25-0.25	0.25-1,000	21.1
Ragweed	2,875	0.38	1.95±14.21	0.25	0.25-0.25	0.25-469	19.3
Oak	2,875	0.38	2.81±19.54	0.25	0.25-0.25	0.25-445	16.4
Thistle	2,874	0.35	1.26±7.31	0.25	0.25-0.25	0.25-275	15.9
Birch	2,875	0.36	2.94±24.99	0.25	0.25-0.25	0.25-879	14.1
*Alternaria*	2,875	0.37	1.56±6.81	0.25	0.25-0.25	0.25-179	13.9
*Aspergillus*	2,875	0.32	0.75±3.01	0.25	0.25-0.25	0.25-62.3	11.7

Some numbers do not add up to the total number of participants because of missing data. sIgE, specific immunoglobulin E; AM, arithmetic mean; SD, standard deviation; Der F, *Dermatophagoides farina*; Der P, *Dermatophagoides pteronyssinus*; GM, geometric mean; LOD, lower limit of detection.

**Table 3.** Association between sIgE^1^ levels and asthma status in children aged 6-19 years (n=2,875)

sIgE	Mode 1^2^			Model 2^3^		
	Never (n=2,401)	Ever (n=474)	Current asthma (n=160)	Never (n=2,401)	Ever (n=474)	Current asthma (n=160)
Der F	Reference	1.17 (1.12, 1.21)^***^	1.22 (1.15, 1.29)^***^	Reference	—	1.11 (1.04, 1.19)^**^
Der P	Reference	1.16 (1.12, 1.21)^***^	1.20 (1.14, 1.27)^***^	Reference	1.09 (1.05, 1.14)^***^	—
Cat	Reference	1.30 (1.22, 1.38)^***^	1.39 (1.29, 1.50)^***^	Reference	—	—
Dog	Reference	1.46 (1.36, 1.57)^***^	1.59 (1.45, 1.74)^***^	Reference	1.25 (1.15, 1.35)^***^	1.30 (1.16, 1.46)^***^
*Alternaria*	Reference	1.30 (1.23, 1.37)^***^	1.39 (1.30, 1.49)^***^	Reference	—	—
*Aspergillus*	Reference	1.52 (1.41, 1.64)^***^	1.75 (1.58, 1.93)^***^	Reference	1.39 (1.28, 1.50)^***^	1.55 (1.39, 1.72)^***^

Values are presented as adjusted odds ratio (95% confidence interval). sIgE, specific immunoglobulin E; ‘—‘, not included in regression model; Der F, *Dermatophagoides farina*; Der P, *Dermatophagoides pteronyssinus*.

1 sIgE was log2 transformed.

2 Adjusted for sex, age, obesity/overweight, and household income.

3 Adjusted for sex, age, household income, obesity/overweight, and all other sIgEs in the table; sIgEs were selected by backward stepwise regression analysis.

** p<0.01,

*** p<0.001.

**Table 4.** Association between sIgE^1^ levels and asthma status in children aged 6-19 years (n=474)

sIgE	Mode 1^2^				Model 2^3^			
	Asthma in remission (n=314)	Current asthma (n=160)	IQR	Per IQR increased^2^	Asthma in remission (n=314)	Current asthma (n=160)	IQR	Per IQR increased^3^
Der F	Reference	1.09 (1.02, 1.17)^*^	3.13	1.31 (1.06, 1.64)	Reference	—		—
Der P	Reference	1.07 (1.00, 1.14)^†^	3.03	1.23 (0.99, 1.49)	Reference	—		—
Cat	Reference	1.15 (1.05, 1.26)^**^	0.86	1.13 (1.04, 1.22)	Reference	1.12 (1.02, 1.24)^*^	0.86	1.10 (1.02, 1.20)
Dog	Reference	1.19 (1.08. 1.32)^***^	1.51	1.30 (1.12, 1.52)	Reference	—		—
*Alternaria*	Reference	1.15 (1.06, 1.25)^**^	1.80	1.29 (1.11, 1.49)	Reference	—		—
*Aspergillus*	Reference	1.22 (1.10, 1.35)^***^	0.82	1.18 (1.08, 1.28)	Reference	1.19 (1.08, 1.33)^***^	0.82	1.15 (1.07, 1.26)

Values are presented as adjusted odds ratio (95% confidence interval). sIgE, specific immunoglobulin E; ‘—‘, not included in regression model; Der F, *Dermatophagoides farina*; Der P, *Dermatophagoides pteronyssinus*; IQR, interquartile range.

1 sIgE was log2 transformed.

2 Adjusted for sex, age, obesity/overweight, and household income.

3 Adjusted for sex, age, household income, obesity/overweight, and all other sIgEs in the table. sIgEs were selected by backward stepwise regression analysis.

† p<0.1,

* p<0.05,

** p<0.01,

*** p<0.001.

## SUPPLEMENTARY MATERIALS

Supplementary Material 1. Specific IgE (sIgE) selection for ever having asthma by bootstrap forest analyzed five times

Supplementary Material 2 The cut point of overweight/obesity in children by sex and age

Supplementary Material 3 Spearmen's correlation between 6 selected specific IgE (sIgE)

Supplementary Material 4 Percentage of under detection limit (%<LOD) of sIgE levels among the three groups Der F, *Dermatophagoides farina*; Der P, *Dermatophagoides pteronyssinus*; sIgE, specific immunoglobulin E.

Supplementary Material 5 Distribution of selected specific IgE (sIgE) levels and asthma status in children, aged 6 to 19 years (n = 2,875)

Supplementary Material 6 Association between sIgE levels and asthma status in children, aged 6 to 19 years (n = 2,875)
