# Supplementary material for: Aspergillus sensitization associated with current asthma in children in the United States: an analysis of data from the 2005-2006 NHANES
Source: Epidemiol Health. 2022 Oct 28;44:e2022099. doi: 10.4178/epih.e2022099 (PMC10185966; doi:10.4178/epih.e2022099)
Supplement: Supplementary Material 1. — Specific IgE (sIgE) selection for ever having asthma by bootstrap forest analyzed five times [file epih-44-e2022099-Supplementary-1.docx]

| **Supplementary Material 1.** Specific IgE (sIgE) selection for ever having asthma by bootstrap forest analyzed five times | | | |
| --- | --- | --- | --- |
| Predictors | Average contribution | Average Portion | Rank |
| ***Aspergillus*** | **28.18** | **0.17** | **1** |
| **Dog** | **26.47** | **0.16** | **2** |
| ***Alternaria*** | **24.68** | **0.15** | **3** |
| **Cat** | **14.12** | **0.09** | **4** |
| **Der F** | **13.98** | **0.08** | **5** |
| **Der P** | **13.43** | **0.08** | **6** |
| Ragweed | 5.32 | 0.03 | 7 |
| Bermuda grass | 5.05 | 0.03 | 8 |
| Rye grass | 4.65 | 0.03 | 9 |
| Thistle | 4.53 | 0.03 | 10 |
| Oak | 4.30 | 0.03 | 11 |
| Birch | 4.26 | 0.03 | 12 |
| Mouse | 3.57 | 0.02 | 13 |
| Peanut | 3.43 | 0.02 | 14 |
| Cockroach | 3.40 | 0.02 | 15 |
| Shrimp | 2.22 | 0.01 | 16 |
| Rat | 1.73 | 0.01 | 17 |
| Milk | 1.62 | 0.01 | 18 |
| Egg | 0.57 | 0.003 | 19 |
| Der F, *Dermatophagoides farina*; Der P, *Dermatophagoides pteronyssinus.* | | | |
