# Supplementary material for: Aspergillus sensitization associated with current asthma in children in the United States: an analysis of data from the 2005-2006 NHANES
Source: Epidemiol Health. 2022 Oct 28;44:e2022099. doi: 10.4178/epih.e2022099 (PMC10185966; doi:10.4178/epih.e2022099)
Supplement: Supplementary Material 2 — The cut point of overweight/obesity in children by sex and age [file epih-44-e2022099-Supplementary-2.docx]

| **Supplementary Material 2**. The cut point of overweight/obesity in children by sex and age | | |
| --- | --- | --- |
| Age | 85th percentile of BMI | |
|  | Boys | Girls |
| 6 | 18.206 | 18.694 |
| 7 | 19.966 | 19.900 |
| 8 | 20.942 | 20.118 |
| 9 | 25.994 | 22.920 |
| 10 | 24.553 | 26.756 |
| 11 | 26.182 | 28.458 |
| 12 | 26.869 | 29.696 |
| 13 | 27.316 | 30.066 |
| 14 | 27.956 | 30.734 |
| 15 | 29.760 | 29.782 |
| 16 | 29.800 | 28.907 |
| 17 | 30.744 | 32.340 |
| 18 | 31.408 | 33.248 |
| 19 | 32.998 | 35.900 |
| Reference:  Defining Childhood Weight Status - BMI for Children and Teens. <https://www.cdc.gov/obesity/basics/childhood-defining.html?CDC_AA_refVal=https%3A%2F%2Fwww.cdc.gov%2Fobesity%2Fchildhood%2Fdefining.html> (Accessed 3 December 2021) | | |
