# Supplementary material for: Aspergillus sensitization associated with current asthma in children in the United States: an analysis of data from the 2005-2006 NHANES
Source: Epidemiol Health. 2022 Oct 28;44:e2022099. doi: 10.4178/epih.e2022099 (PMC10185966; doi:10.4178/epih.e2022099)
Supplement: Supplementary Material 3 — Spearmen's correlation between 6 selected specific IgE (sIgE) [file epih-44-e2022099-Supplementary-3.docx]

| **Supplementary Material 3.** Spearmen's correlation between 6 selected specific IgE (sIgE) | | | | | | |
| --- | --- | --- | --- | --- | --- | --- |
| sIgE | Der F | Der P | Cat | Dog | *Alternaria* | *Aspergillus* |
| Der F | ⎯ |  |  |  |  |  |
| Der P | 0.920*** | ⎯ |  |  |  |  |
| Cat | 0.308*** | 0.319*** | ⎯ |  |  |  |
| Dog | 0.399*** | 0.423*** | 0.657*** |  |  |  |
| *Alternaria* | 0.159*** | 0.187*** | 0.371*** | 0.281*** | ⎯ |  |
| *Aspergillus* | 0.196*** | 0.229*** | 0.403*** | 0.298*** | 0.747*** | ⎯ |
| ***p value <0.0001.  Der F, *Dermatophagoides farina*; Der P, *Dermatophagoides pteronyssinus.* | | | | | | |
