# Supplementary material for: Aspergillus sensitization associated with current asthma in children in the United States: an analysis of data from the 2005-2006 NHANES
Source: Epidemiol Health. 2022 Oct 28;44:e2022099. doi: 10.4178/epih.e2022099 (PMC10185966; doi:10.4178/epih.e2022099)
Supplement: Supplementary Material 4 — Percentage of under detection limit (%<LOD) of sIgE levels among the three groups Der F, Dermatophagoides farina; Der P, Dermatophagoides pteronyssinus; sIgE, specific immunoglobulin E. [file epih-44-e2022099-Supplementary-4.docx]

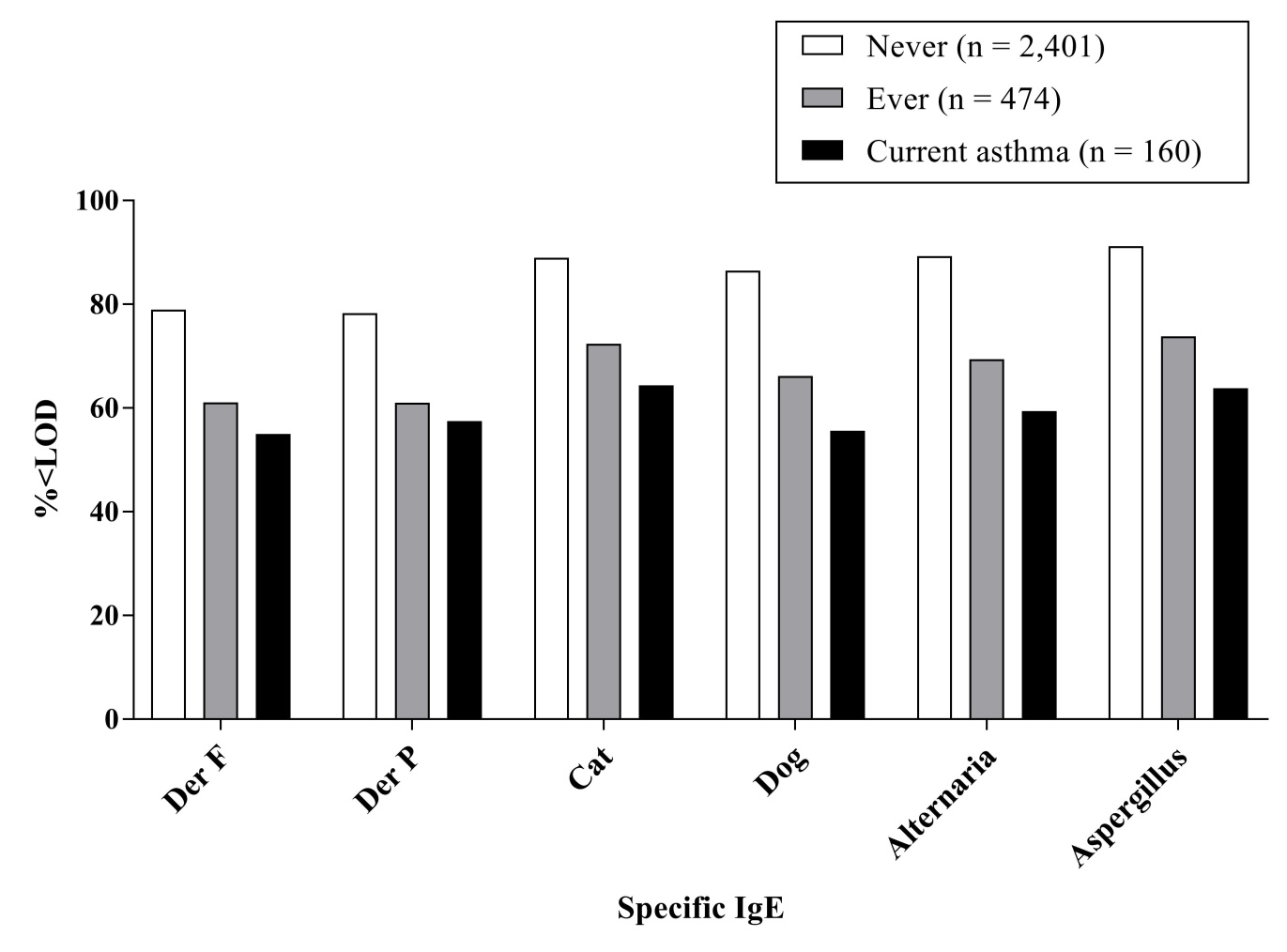


**Supplementary Material 4.** Percentage of under detection limit (%<LOD) of sIgE levels among the three groups

Der F, *Dermatophagoides farina*; Der P, *Dermatophagoides pteronyssinus*; sIgE, specific immunoglobulin E.
