# Supplementary material for: Aspergillus sensitization associated with current asthma in children in the United States: an analysis of data from the 2005-2006 NHANES
Source: Epidemiol Health. 2022 Oct 28;44:e2022099. doi: 10.4178/epih.e2022099 (PMC10185966; doi:10.4178/epih.e2022099)
Supplement: Supplementary Material 5 — Distribution of selected specific IgE (sIgE) levels and asthma status in children, aged 6 to 19 years (n = 2,875) [file epih-44-e2022099-Supplementary-5.docx]

| **Supplementary Material 5.** Distribution of selected specific IgE (sIgE) levels and asthma status in children, aged 6 to 19 years (n = 2,875) | | | | | | | | | | | |
| --- | --- | --- | --- | --- | --- | --- | --- | --- | --- | --- | --- |
| sIgE | Never  (n = 2,401) | |  | Asthma | | | | |  | P-value | |
|  |  |  |  | Ever (n = 474) | |  | Current  (n = 160) | |  | Never v.s. Ever | Never v.s. Current |
|  | Mean (SD) | GM |  | Mean (SD) | GM |  | Mean (SD) | GM |  |  |  |
| **Crude concentration** | |  |  |  |  |  |  |  |  |  |  |
| Der F | 5.23 (40.25) | 0.45 |  | 10.38 (37.56) | 0.84 |  | 13.79 (45.22) | 1.08 |  | <0.0001 | <0.0001 |
| Der P | 5.67 (40.66) | 0.46 |  | 13.64 (53.72) | 0.87 |  | 15.91 (53.72) | 1.06 |  | <0.0001 | <0.0001 |
| Cat | 1.25 (12.88) | 0.32 |  | 4.09 (20.79) | 0.50 |  | 6.15 (21.09) | 0.67 |  | <0.0001 | <0.0001 |
| Dog | 0.58 (2.81) | 0.30 |  | 4.51 (30.88) | 0.51 |  | 8.48 (48.29) | 0.69 |  | <0.0001 | <0.0001 |
| *Alternaria* | 1.10 (4.67) | 0.33 |  | 3.88 (12.81) | 0.61 |  | 4.98 (12.11) | 0.82 |  | <0.0001 | <0.0001 |
| *Aspergillus* | 0.46 (1.30) | 0.29 |  | 2.19 (6.63) | 0.49 |  | 3.16 (8.57) | 0.67 |  | <0.0001 | <0.0001 |
|  |  |  |  |  |  |  |  |  |  |  |  |
| **Log2 transformed** | |  |  |  |  |  |  |  |  |  |  |
| Der F | -1.17 (2.00) |  |  | -0.26 (2.74) |  |  | 0.12 (2.97) |  |  | <0.0001 | <0.0001 |
| Der P | -1.12 (2.06) |  |  | -0.19 (2.86) |  |  | 0.08 (3.05) |  |  | <0.0001 | <0.0001 |
| Cat | -1.67 (1.17) |  |  | -1.00 (2.06) |  |  | -0.57 (2.46) |  |  | <0.0001 | <0.0001 |
| Dog | -1.72 (0.92) |  |  | -0.96 (1.95) |  |  | -0.53 (2.31) |  |  | <0.0001 | <0.0001 |
| *Alternaria* | -1.59 (1.34) |  |  | -0.71 (2.27) |  |  | -0.29 (2.51) |  |  | <0.0001 | <0.0001 |
| *Aspergillus* | -1.77 (0.85) |  |  | -1.02 (1.90) |  |  | -0.58 (2.18) |  |  | <0.0001 | <0.0001 |
| P value was calculated by Kruskal-Wallis test. | | | | | | | | | | | |
| GM, geometrical mean; Der F, *Dermatophagoides farina*; Der P, *Dermatophagoides pteronyssinus*; SD, standard deviation. | | | | | | | | | | | |
