# Supplementary material for: Aspergillus sensitization associated with current asthma in children in the United States: an analysis of data from the 2005-2006 NHANES
Source: Epidemiol Health. 2022 Oct 28;44:e2022099. doi: 10.4178/epih.e2022099 (PMC10185966; doi:10.4178/epih.e2022099)
Supplement: Supplementary Material 6 — Association between sIgE levels and asthma status in children, aged 6 to 19 years (n = 2,875) [file epih-44-e2022099-Supplementary-6.docx]

| **Supplementary Material 6.** Association between sIgE levels and asthma status in children, aged 6 to 19 years (n = 2,875) | | | | | | |
| --- | --- | --- | --- | --- | --- | --- |
| sIgEs | OR1 (95%CI) | | |  | OR2 (95%CI) | |
|  | Never  (n = 2,401) | Ever (n = 474) | Current asthma  (n = 160) |  | Asthma in remission  (n = 314) | Current asthma (n = 160) |
| Der F | Reference | 1.17 (1.13-1.21)*** | 1.22 (1.15-1.28)*** |  | Reference | 1.08 (1.01-1.15)* |
| Der P | Reference | 1.16 (1.12-1.21)*** | 1.19 (1.13-1.26)*** |  | Reference | 1.05 (0.98-1.12) |
| Cat | Reference | 1.29 (1.22-1.37)*** | 1.39 (1.29-1.49)*** |  | Reference | 1.15 (1.06-1.26)** |
| Dog | Reference | 1.47 (1.37-1.58)*** | 1.60 (1.46-1.75)*** |  | Reference | 1.18 (1.07-1.29)** |
| *Alternaria* | Reference | 1.31 (1.24-1.37)*** | 1.40 (1.31-1.50)*** |  | Reference | 1.12 (1.04-1.22)** |
| *Aspergillus* | Reference | 1.52 (1.41-1.63)*** | 1.71 (1.56-1.88)*** |  | Reference | 1.19 (1.08-1.31)*** |
| sIgE was log 2 transformed. | | | | | | |
| #p<0.1; *p<0.05; **p<0.01; ***p<0.001 | | | | | | |
| Der F, *Dermatophagoides farina*; Der P, *Dermatophagoides pteronyssinus*; OR, odds ratio. | | | | | | |
